# Huangqi-Danshen decoction protects against cisplatin-induced acute kidney injury in mice

**DOI:** 10.3389/fphar.2023.1236820

**Published:** 2023-11-16

**Authors:** Xinhui Liu, Liwen Gao, Xi Huang, Ruyu Deng, Shanshan Wu, Yu Peng, Jiandong Lu

**Affiliations:** ^1^ Department of Nephrology, Shenzhen Traditional Chinese Medicine Hospital, Guangzhou University of Chinese Medicine, Shenzhen, Guangdong, China; ^2^ The Fourth Clinical Medical College, Guangzhou University of Chinese Medicine, Shenzhen, Guangdong, China; ^3^ Shenzhen Traditional Chinese Medicine Hospital Affiliated to Nanjing University of Chinese Medicine, Shenzhen, Guangdong, China

**Keywords:** acute kidney injury, Huangqi-Danshen decoction, apoptosis, inflammation, oxidative stress, metabolomics, nicotinamide adenine dinucleotide

## Abstract

**Background:** Acute kidney injury (AKI) induced by cisplatin remains a major impediment to the clinical application of cisplatin, necessitating urgent exploration for promising solutions. Huangqi-Danshen decoction (HDD), a Chinese herbal preparation, has been shown by our group to have a reno-protective effect in adenine-induced chronic kidney disease mice and diabetic *db/db* mice. However, the effect of HDD on cisplatin-induced AKI and its underlying mechanisms are unknown.

**Methods:** The AKI model was established by intraperitoneal injection of cisplatin (20 mg/kg) in C57BL/6 mice. The mice in the treatment group were administrated with HDD (6.8 g/kg/d) for 5 consecutive days before cisplatin challenge. After 72 h cisplatin injection, blood and kidney tissue were subsequently collected for biochemical detection, histopathological evaluation, Western blot analysis, immunohistochemical staining, and terminal deoxynucleotidyl transferase (TdT)-mediated dUTP nick end labeling assay. Ultra-high-performance liquid chromatography coupled with quadrupole time-of-flight mass spectrometry was used to detect changes in renal metabolites.

**Results:** The results showed that HDD significantly reduced serum creatinine and blood urea nitrogen levels and alleviated renal histopathological injury in cisplatin-induced AKI mice. And HDD treatment demonstrated a significant inhibition in apoptosis, inflammation, and oxidative stress in AKI mice. Moreover, non-target metabolomics revealed that HDD significantly restored 165 altered metabolites in AKI mice. Subsequent enrichment analysis and pathway analysis of these metabolites indicated that nicotinate and nicotinamide metabolism was the primary pathway affected by HDD intervention. Further investigation showed that HDD could upregulate nicotinamide adenine dinucleotide (NAD^+^) biosynthesis-related enzymes quinolinate phosphoribosyltransferase, nicotinamide mononucleotide adenylyltransferase 1, and nicotinamide phosphoribosyltransferase to replenish NAD^+^ content in the kidney of AKI mice.

**Conclusion:** In summary, HDD exerted a protective effect against cisplatin-induced AKI and suppressed apoptosis, inflammation, and oxidative stress in the kidney of AKI mice, which may be attributed to the modulation of NAD^+^ biosynthesis.

## Introduction

Acute kidney injury (AKI) is a sharp decrease in glomerular filtration function over a short period of time due to various aetiologies (Hoste et al., 2018). AKI is associated with considerable morbidity and mortality and has a high risk of development of chronic kidney disease (CKD) or end-stage kidney disease (ESKD) (Lameire et al., 2013). Cisplatin [*cis*-diamminedichloroplatinum (II)], an inorganic platinum derivative, is widely used to treat solid tumors including those of the head, neck, lung, breast, ovary, and testis (Dasari and Tchounwou, 2014; Zhang et al., 2021). Cisplatin is mainly cleared by the kidneys and therefore has a high risk of causing acute and chronic nephrotoxicity. It was reported that AKI occurred in 31.5% of patients with cancer who received cisplatin for treatment across multiple tumor types (Latcha et al., 2016). AKI is the dose-limiting side effect of cisplatin that limits its clinical application in cancer patients (Karasawa and Steyger, 2015). Therefore, it is urgent to further explore the underlying mechanism and effective therapeutic regimens of cisplatin-induced AKI.

In recent years, accumulating evidence elucidates the reno-protective effect of Chinese herbal medicine (CHM) in multiple AKI models via different mechanisms of inhibiting inflammation, cell apoptosis, necroptosis, ferroptosis, and restraining oxidative stress etc., (Li H. D. et al., 2019; Li J. et al., 2023). Chen *et al.* included 15 randomized controlled trials to evaluate the efficacy of CHM as an adjunctive therapy for patients with AKI. The results showed that patients randomly assigned to CHM plus Western treatment had a statistically significant reduction in in-hospital mortality compared with those randomly assigned to Western treatment alone (Chen et al., 2016). In traditional Chinese medicine (TCM) theory, AKI is caused by qi deficiency, blood stasis, and toxin accumulation. Huangqi-Danshen decoction (HDD) is composed of Astragalus membranaceus (Huangqi) and Salvia miltiorrhiza (Danshen) and has the effect of invigorating qi and promoting blood circulation. According to TCM theory, HDD would be beneficial in treating AKI. Our previous studies have found that HDD had reno-protective effect in adenine-induced CKD mice and *db/db* diabetic mice (Liu et al., 2019a; Liu et al., 2019b; Liu et al., 2020). However, the protective effects and underlying mechanisms of HDD against cisplatin-induced AKI are unknown.

Metabolomics provides a tool to qualitatively and quantitatively analyze small molecular metabolites (typically <1,500 Da) in specific tissues, organs or biological fluids (German et al., 2005). Metabolomics, with its high-throughput and high-resolution characteristics, allows accurate analysis of metabolite changes in body fluids or tissues, thus helping to understand the development and progression of diseases (Abbiss et al., 2019). Several studies have used metabolomics analysis to identify novel biomarkers for the development, progression and prognosis of AKI and to reveal specific mechanisms of AKI (Cui et al., 2021; Ping et al., 2021; Tan et al., 2021; Yuan et al., 2022). Metabolomics reflects the overall changes of the organism by studying the changes of metabolites, which is similar to the holistic view of TCM and provides new ideas and methods for the research of CHM. The application of metabonomics is helpful to further clarify the mechanism of action of CHM (Wang et al., 2017; Wang et al., 2021). In this study, we first evaluated the protective effect of HDD on cisplatin-induced AKI, and secondly, we used ultra-high-performance liquid chromatography coupled with quadrupole time-of-flight mass spectrometry (UHPLC-QTOF/MS) to detect changes in renal metabolites to clarify the mechanism of action of HDD.

## Materials and methods

### Chemicals and antibodies

Cisplatin was purchased from Sigma-Aldrich (St. Louis, MO, United States). The primary antibodies used in this study were neutrophil gelatinase-associated lipocalin (NGAL), Bax, 4-hydroxynonenal (4-HNE), nicotinamide mononucleotide adenylyltransferase 1 (NMNAT1) (Abcam, Cambridge, MA, United States); nicotinamide phosphoribosyltransferase (NAMPT) (Proteintech, Wuhan, China); 8-hydroxy-2′-deoxyguanosine (8-OHdG) (Santa CruzBiotechnology, Santa Cruz, CA, United States); cleaved caspase-3, F4/80, p53, p-p53 (Cell Signaling Technology, Beverly, MA, United States); quinolinic acidphosphoribosyltransferase (QPRT), and β-actin (Sigma-Aldrich, St Louis, MO, United States). The horseradish peroxidase (HRP)-conjugated secondary antibodies were obtained from Thermo Fisher Scientific (Waltham, MA, United States).

### HDD preparation

HDD was composed of 2 herbs: *Astragalus mongholicus* Bunge [Fabaceae] and *Salvia miltiorrhiza* Bunge [Lamiaceae], in the ratio of 2:1. These two herbs were extracted twice with 8 times of ddH_2_O (volume/weight) for 1 h each time. The extracts obtained twice were mixed together, filtered and concentrated to obtain an HDD extract with a final concentration of 1 g of raw herbs per mL of extract. High performance liquid chromatography-mass spectrometry (HPLC-MS) analysis was conducted to confirm the quality of HDD extract (Supplementary Figure S1).

### Animals and treatment

Eighteen male C57BL/6 mice (6–8 weeks old) were purchased from Guangdong Medical Laboratory Animal Center (Foshan, China). After 1 week of adaptive feeding, all mice were randomly divided into 3 groups: the control group (*n* = 6), the AKI group (*n* = 6), and the AKI + HDD group (*n* = 6). The AKI mice received single intraperitoneal injection of cisplatin (20 mg/kg) (Yu et al., 2018), while AKI + HDD group was pretreated with HDD at the dose of 6.8 g/kg/d for 5 consecutive days before cisplatin challenge. After 72 h cisplatin injection, all mice were sacrificed. After clotting, the blood was centrifuged at 1,500 rpm for 10 min at 4°C to isolate serum. A portion of kidneys were fixed with 4% paraformaldehyde for pathological staining, and the remaining kidneys were frozen at −80°C for protein expression analysis.

### Biochemical assay

The serum creatinine (Scr) and blood urea nitrogen (BUN) concentrations were measured by using specific kits (StressMarq Biosciences, British Columbia, Canada) in accordance with manufacturer’s instructions.

### Histological assessment

To evaluate renal pathological injury, periodic acid-Schiff (PAS) staining was conducted on paraffin-embedded kidney sections. Quantitative analysis of tubular injury was performed according to the following scoring criteria: normal tubules = 0; less than 25% tubular injury = 1; 25%–50% tubular injury = 2; 50%–75% tubular injury = 3; more than 75% tubular injury = 4 (Weidemann et al., 2008).

### Western blotting

Kidney tissues were homogenized in RIPA lysis buffer on ice. Then, the supernatants were separated and the protein concentrations were determined by Bradford method. Equal amounts of protein were electrophoresed in 10% or 15% SDS-PAGE gels, transferred to nitrocellulose or polyvinylidene difluoride membranes, and blocked with 5% non-fat milk. Next, these membranes were incubated with the corresponding primary and secondary antibodies. To visualize protein bands, these membranes were treated with ECL luminescent solution. The gray values of protein bands were calculated by Image Lab software version 5.1 (Bio-Rad Laboratories, Hercules, CA, United States).

### Terminal deoxynucleotidyl transferase (TdT)-mediated dUTP nick end labeling (TUNEL)

Detection of apoptotic cells in paraffin-embedded kidney sections was performed by using One Step TUNEL Apoptosis Assay Kit (Beyotime, Shanghai, China). In brief, kidney tissue sections were dewaxed, hydrated, and treated with DNase-free proteinase K. After washing with PBS, the sections were incubated with TUNEL detection solution at 37°C for 60 min in the dark. The numbers of TUNEL positive cells per field were counted under ×200 microscopic fields.

### Immunohistochemistry

Paraffin-embedded kidney tissues were cut into 6 µm sections. After deparaffinization and hydration, the sections were sequentially treated with citrate antigen retrieval solution, 3% hydrogen peroxide, and goat serum. Then, the sections were incubated with primary antibodies against F4/80, 4-HNE, and 8-OHdG at 4°C overnight. The brown positive staining area was visualized by using diaminobenzidine (DAB) solution. The numbers of F4/80 positive cells per field were counted under ×400 microscopic fields. The integrated optical density (IOD) values of 4-HNE and 8-OHdG positive staining areas were calculated by ImagePro Plus 6.0 software (Media Cybernetics, CA, United States).

### UHPLC-QTOF/MS analysis

A 25 mg kidney tissue sample was grinded with 1 mL of extract solution (methanol: acetonitrile: water = 2:2:1). After centrifugation at 10,000 rpm for 15 min at 4°C, the supernatant was collected for vacuum drying. Then, the dried samples were reconstituted with 200 μL of 50% acetonitrile and centrifuged at 13,000 rpm for 15 min at 4°C. The supernatant was used for on-board detection. Chromatographic separation was carried out on Agilent 1290 UHPLC system (Agilent Technologies, Santa Clara, CA, United States). The chromatographic parameters were: column, BEH Amide (2.1 × 100 mm, 1.7 μm, Waters, Milford, MA, United States); mobile phase, 25 mmol/L ammonium acetate and 25 mmol/L ammonia (A) and acetonitrile (B); gradient elution conditions, 0–0.5 min, 95%B; 0.5–7.0 min, 95%–65% B; 7.0–8.0 min, 65%–40% B; 8.0–9.0 min, 40% B; 9.0–9.1 min, 40%–95% B; 9.1–12.0 min, 95% B; column temperature: 25°C; auto-sampler temperature, 4°C; injection volume, 1 μL. MS spectra were acquired using TripleTOF 6,600 mass spectrometry (AB Sciex, Framingham, MA, United States). The electrospray ionization interface (ESI) source conditions were: source temperature, 600°C; gas 1, 60 psi; gas 2, 60 psi; curtain gas, 35 psi; declustering potential, 60 V; ion spray voltage floating, 5 kV (pos) or −4 kV (neg).

The raw data were processed by R package XCMS (version 3.2), including retention time correction, peak identification, peak extraction, peak integration, peak alignment, etc. Then, the data matrices were imported into MetaboAnalyst for statistical and pathway analysis. Principal component analysis (PCA), partial least squares-discriminant analysis (PLS-DA), sparse partial least squares-discriminant analysis (sPLS-DA), and orthogonal partial least squares-discriminant analysis (orthoPLS-DA) were used to show differences in metabolite profiles between groups. Pathway analysis was performed based on Kyoto Encyclopedia of Genes and Genomes (KEGG).

### Statistical analysis

Data were expressed as mean ± standard error of mean (SEM). Statistical differences were calculated using one-way ANOVA, and the method for multiple comparisons was Tukey (SPSS 16.0, Chicago, IL, United States). *p* < 0.05 was considered statistically significant.

## Results

### HDD attenuated cisplatin-induced AKI in mice

**FIGURE 1:**
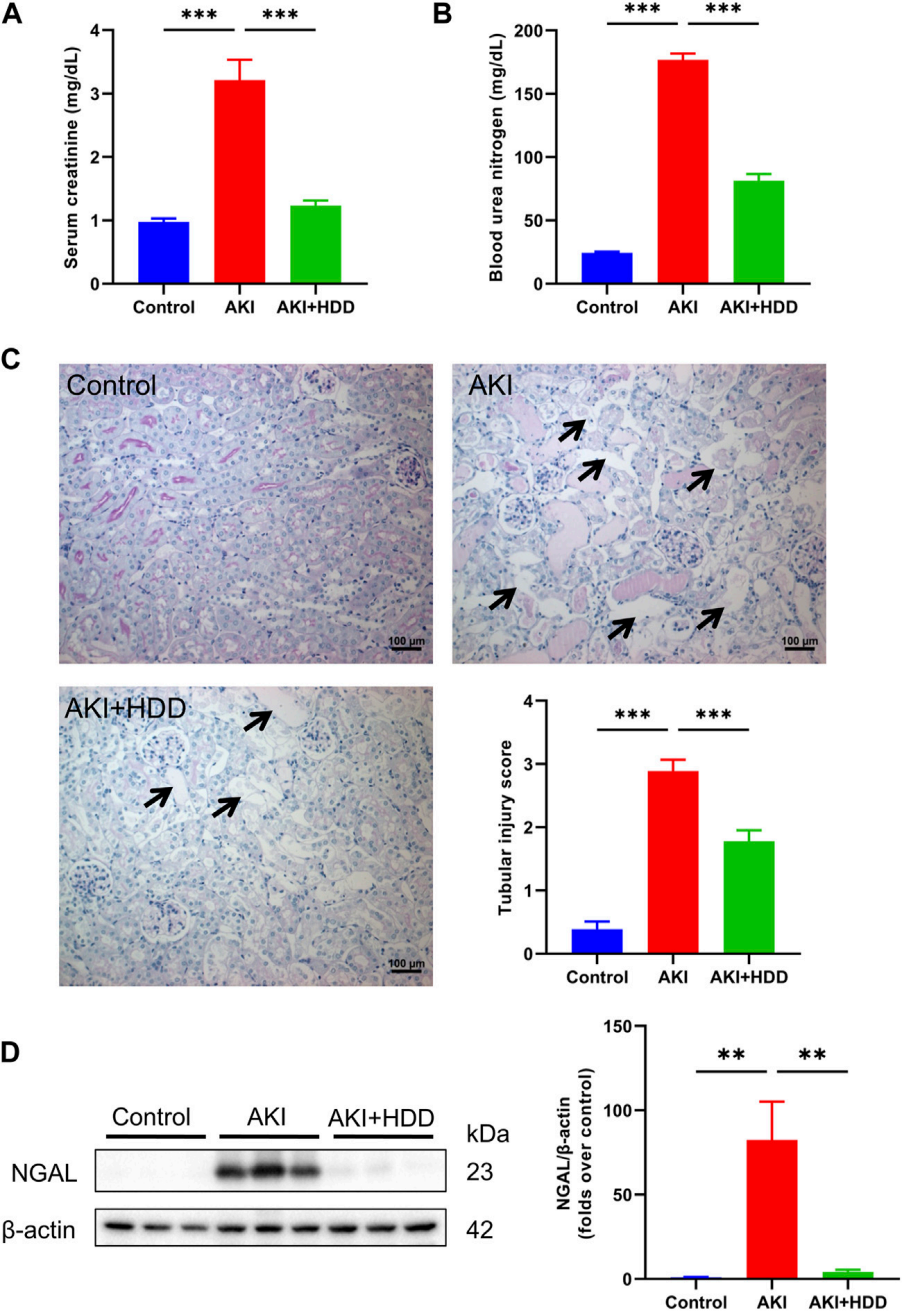
Effects of HDD on cisplatin-induced acute kidney injury in mice. **(A)** Serum creatinine levels. **(B)** Blood urea nitrogen levels. **(C)** Representative images of renal PAS staining (scale bars = 100 μm, magnification = ×200, arrows indicate tubule degeneration) and quantitative analysis of renal tubular injury. **(D)** Western blot analysis of NGAL expression in the kidneys of each group. Data are expressed as mean ± SEM, *n* = 6 mice per group, one-way ANOVA followed by Tukey’s *post hoc* test was used for calculating statistical differences, ^**^ *p* < 0.01, ^***^ *p* < 0.001.

AKI is characterized by a dramatic decline in kidney function over a short period of time, resulting in elevated Scr and BUN levels. In this study, the levels of Scr and BUN of model mice increased 3-fold and 7-fold, respectively, after 3 days of cisplatin challenge. Pretreatment with HDD could prevent the significant elevation of Scr and BUN in AKI mice (*p* < 0.001, Figures 1A, B). PAS staining indicated obvious tubular injury in AKI mice, including tubular epithelial cell necrosis, shedding, and tubular dilation. In contrast, these tubular injuries were significantly attenuated in mice pretreated with HDD (Figure 1C). In addition, the expression of NGAL, a renal tubular injury marker, was strikingly increased in the kidney of AKI mice, and was significantly blunted after HDD pretreatment (Figure 1D). Collectively, these data demonstrated that HDD protected against cisplatin-induced AKI in mice.

### HDD decreased apoptosis in AKI mice

**FIGURE 2:**
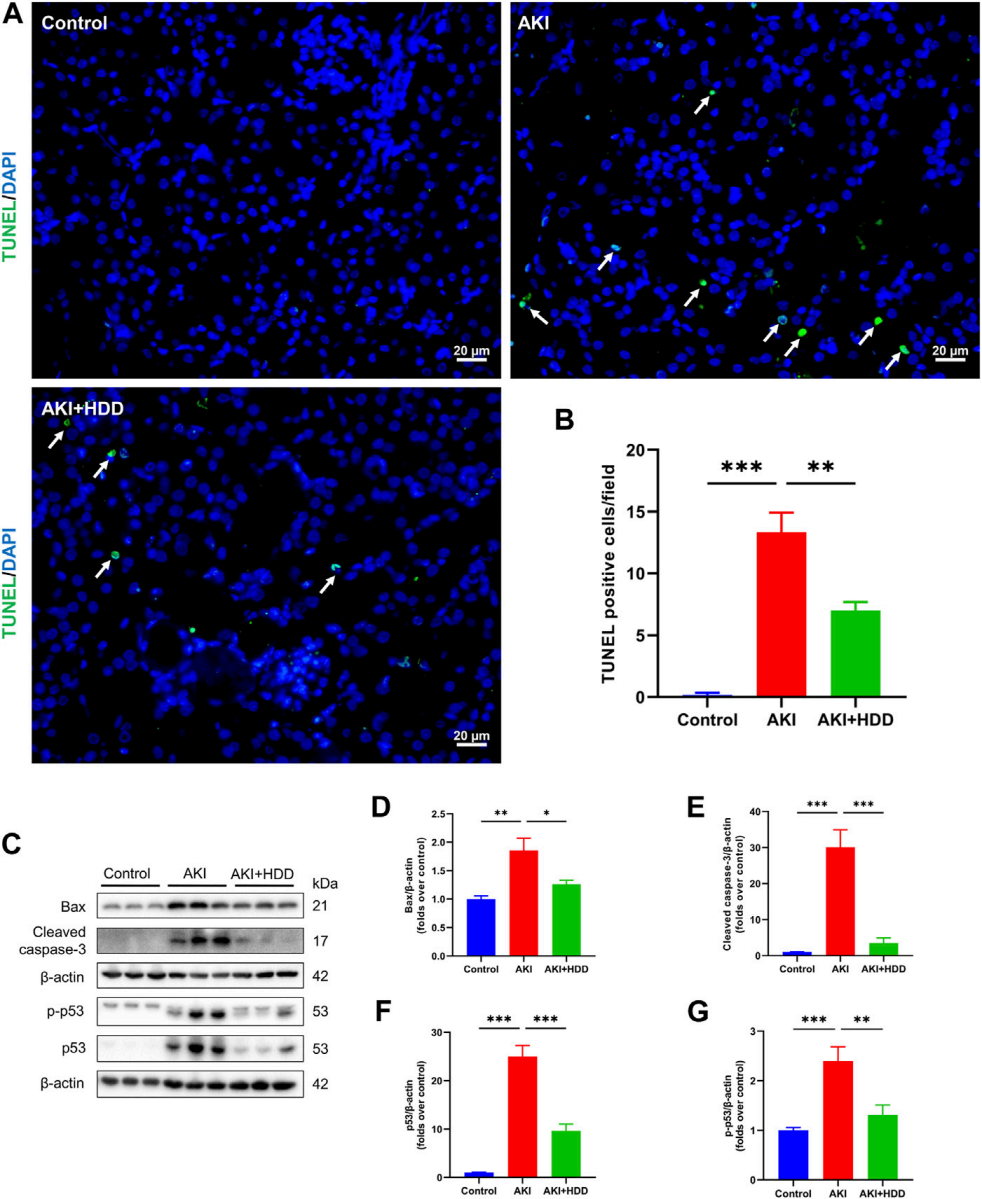
Effects of HDD on apoptosis in AKI mice. **(A)** Representative images of TUNEL staining of apoptotic cells in the kidney of each group (scale bars = 20 μm, magnification = ×400, arrows indicate apoptotic tubular cells). **(B)** Quantitative analysis of TUNEL-positive cells. **(C)** Representative Western blot images of Bax, cleaved caspase-3, p53, and p-p53 expression in the kidneys of each group. **(D–G)** Densitometric analysis of Bax, cleaved caspase-3, p53, and p-p53 expression, normalized to β-actin. Data are expressed as mean ± SEM, *n* = 6 mice per group, one-way ANOVA followed by Tukey’s *post hoc* test was used for calculating statistical differences, ^*^ *p* < 0.05, ^**^ *p* < 0.01, ^***^ *p* < 0.001.

Renal tubular epithelial cell apoptosis was detected through TUNEL staining. As shown in Figures 2A, B, there were rare TUNEL-positive cells in the kidneys of mice in the control group. The number of TUNEL-positive cells was increased significantly in the kidney of AKI mice, which was notably decreased by HDD pretreatment. Moreover, Western blot analysis showed that the enhanced protein expressions of Bax, cleaved caspase-3, p53, and p-p53 in the kidneys of AKI mice were markedly suppressed by HDD pretreatment (Figures 2C–G). These findings suggested that HDD decreased apoptosis in cisplatin-induced AKI mice.

### HDD alleviated inflammation and oxidative stress in AKI mice

**FIGURE 3:**
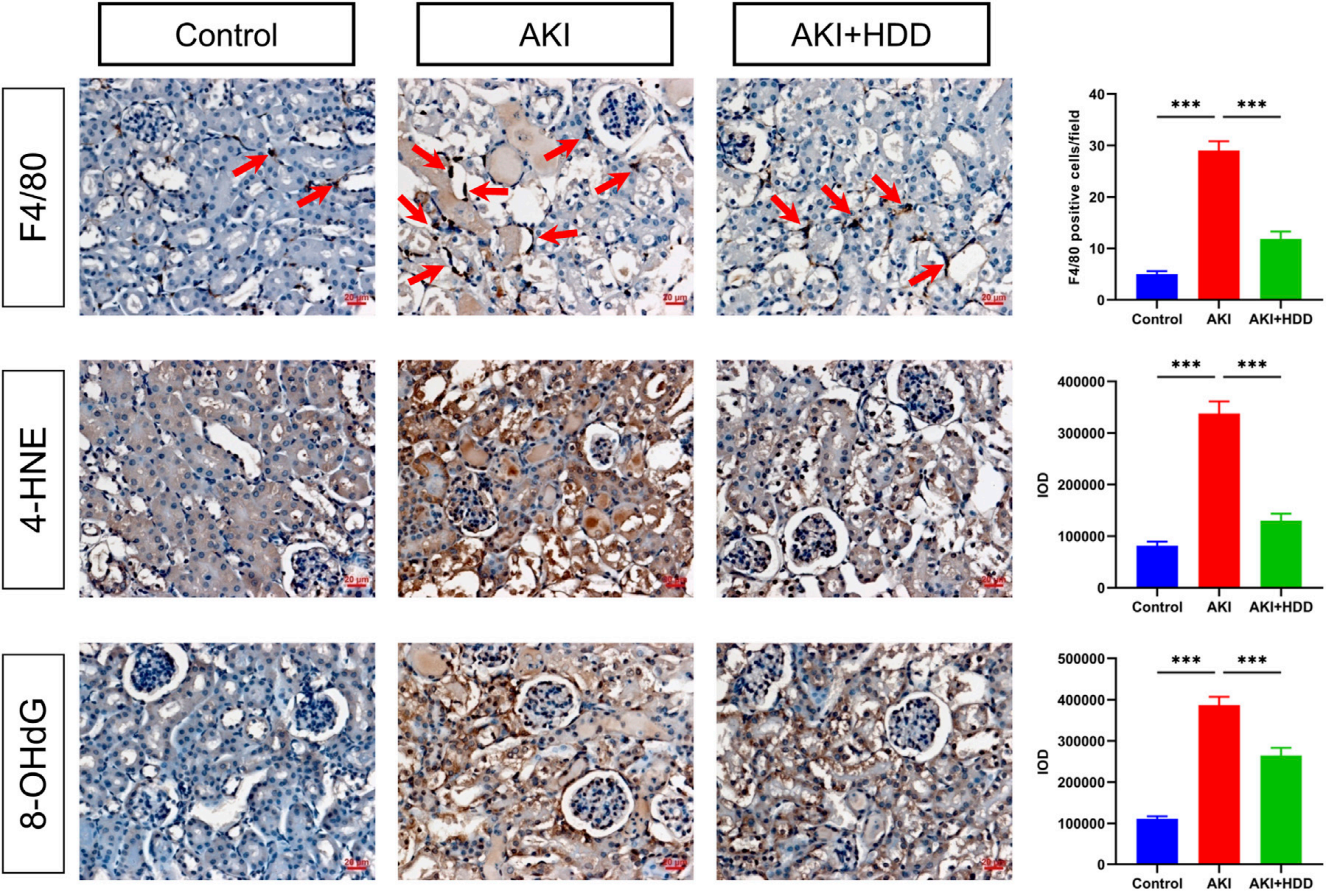
Effects of HDD on inflammation and oxidative stress in AKI mice. Immunohistochemical staining and quantitative analysis of F4/80, 4-HNE, and 8-OHdG in the kidneys of each group. Scale bars = 20 μm, magnification = ×400, arrows indicate F4/80-positive cells. Data are expressed as mean ± SEM, *n* = 3 mice per group, one-way ANOVA followed by Tukey’s *post hoc* test was used for calculating statistical differences, ^***^ *p* < 0.001.

Inflammatory response and oxidative stress are significant pathological features of cisplatin-induced AKI (McSweeney et al., 2021). IHC staining of F4/80, a macrophage marker, revealed that cisplatin injection significantly increased the number of F4/80-positive cells in the mouse kidney, which was reduced by HDD administration (Figure 3). Oxidative stress was assessed by lipid peroxidation product 4-HNE and DNA oxidation marker 8-OHdG. As shown in Figure 3, cisplatin-induced increase of 4-HNE and 8-OHdG in mice kidneys was reduced by 61.5% and 31.7% (*p* < 0.001), respectively, after HDD pretreatment. These data suggested the anti-inflammatory and anti-oxidative stress effects of HDD in cisplatin-induced AKI mice.

### HDD regulated renal metabolite profiles in AKI mice

**FIGURE 4:**
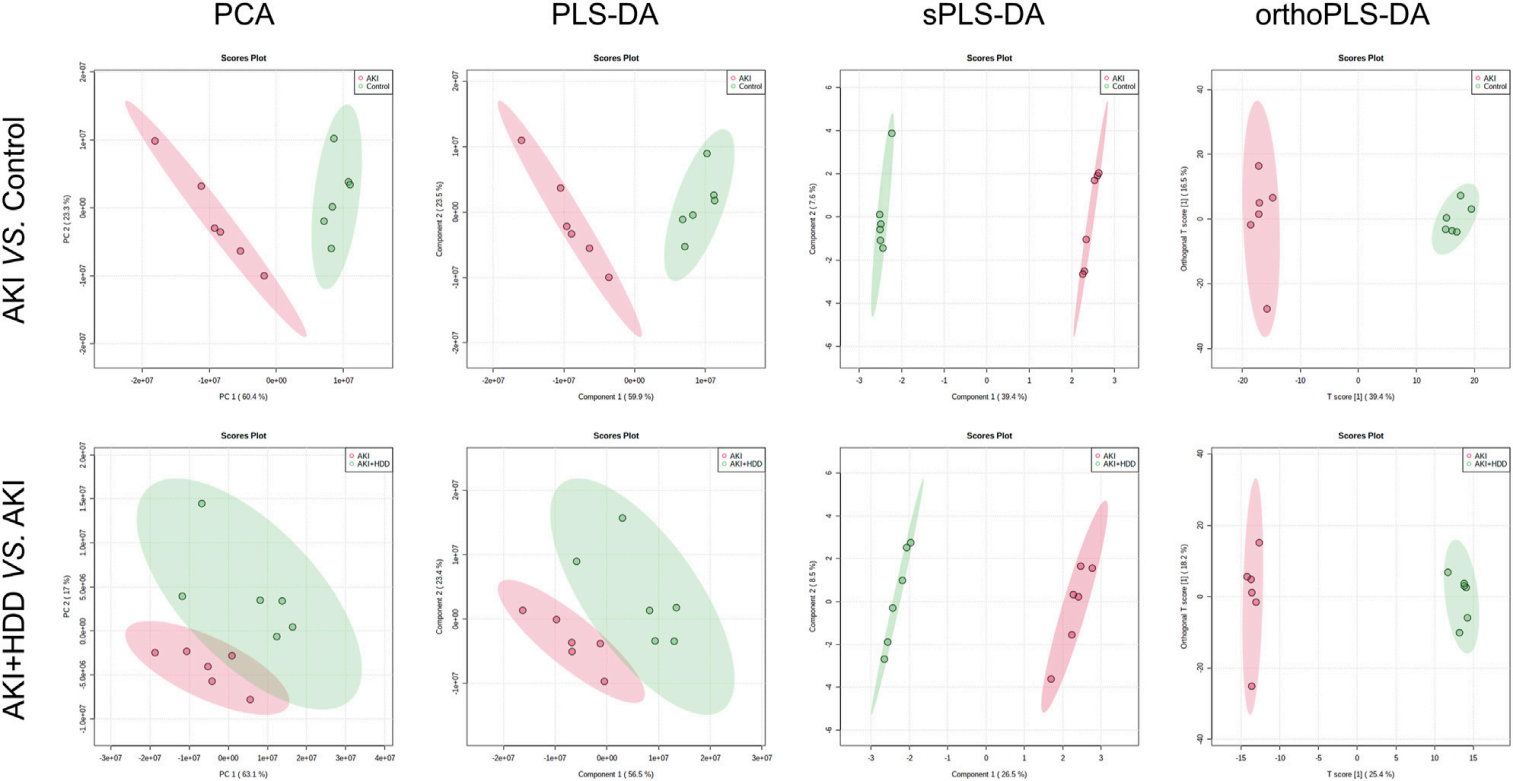
Effects of HDD on renal metabolic profile in AKI mice. PCA, PLS-DA, sPLS-DA, and orthoPLS-DA analyses showed differences in renal metabolic profiles when comparing AKI to control and AKI + HDD to AKI.

**FIGURE 5:**
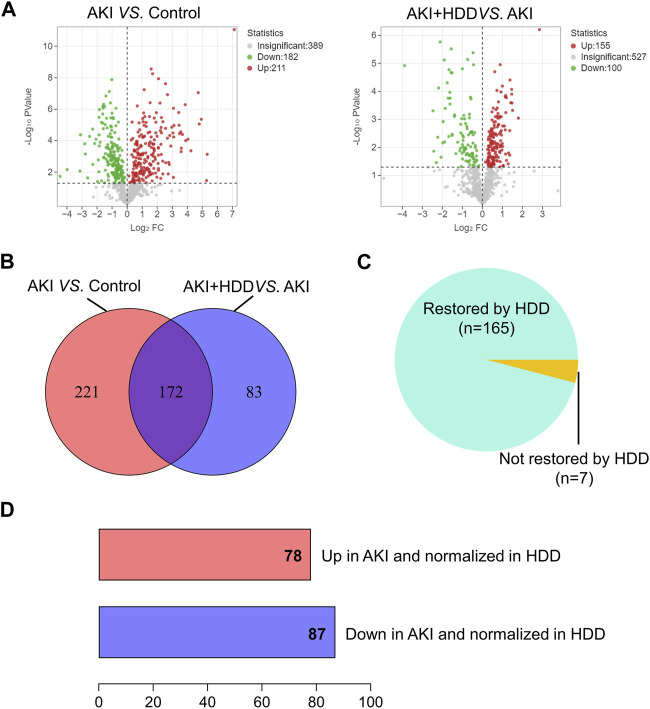
Identification of significantly restored metabolites by HDD in AKI mice. **(A)** Volcano plots of metabolite distribution when comparing AKI to control and AKI + HDD to AKI. **(B)** Venn diagram of the significantly altered metabolites after comparison. **(C)** Pie chart of the proportion of metabolites that could be restored by HDD or not in AKI mice. **(D)** Numbers of metabolites upregulated and downregulated in AKI and normalized in HDD.

**FIGURE 6:**
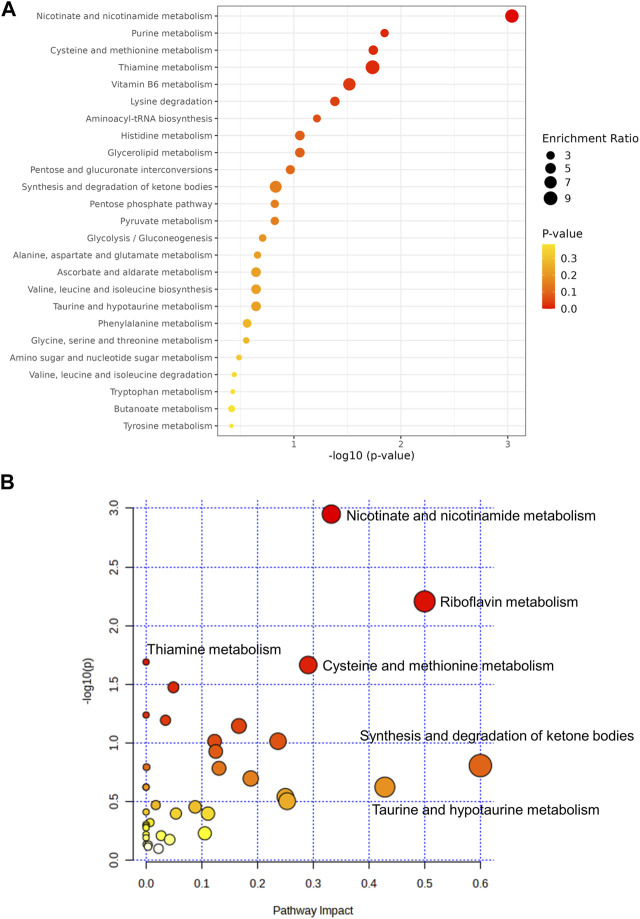
Analysis of the 165 metabolitesrestored by HDD. **(A)** Enrichment analysis. **(B)** Pathway analysis.

To further explore the underlying mechanism by which HDD protected against AKI, we performed non-target metabolomics to detect changes in renal metabolites. In multivariate analysis, there was a clear metabolites separation between the AKI and the control group in PCA, PLS-DA, sPLS-DA, and orthoPLS-DA models. Metabolites in the AKI + HDD and AKI groups had partial overlap in the PCA and PLS-DA models, while they were completely separated in the sPLS-DA and orthoPLS-DA models (Figure 4). Compared to controls, cisplatin challenge significantly increased 211 and decreased 182 metabolites in the kidneys of mice. Administration of HDD regulated renal metabolite profiles by upregulating 155 and downregulating 100 metabolites in AKI mice (Figure 5A). Comparing the metabolites that changed significantly in the two comparison groups yielded 172 overlapping metabolites (Figure 5B), 165 of which could be reversed by HDD treatment and 7 of which did not respond to HDD treatment (Figure 5C). Of the 165 metabolites normalized by HDD, 78 were upregulated and 87 were downregulated in AKI (Figure 5D). Details of these 165 metaboliteswere summarized in Supplementary Table S1. Both enrichment analysis and pathway analysis of these 165 metabolites that responded to HDD treatment revealed that nicotinate and nicotinamide metabolism was the main pathway that was significantly altered (Figure 6). Taken together, these data indicated that HDD could regulate renal metabolite profiles in AKI mice, with a primary focus on nicotinate and nicotinamide metabolism.

### HDD corrected disturbed nicotinamide adenine dinucleotide (NAD^+^) metabolism in AKI mouse kidneys

**FIGURE 7:**
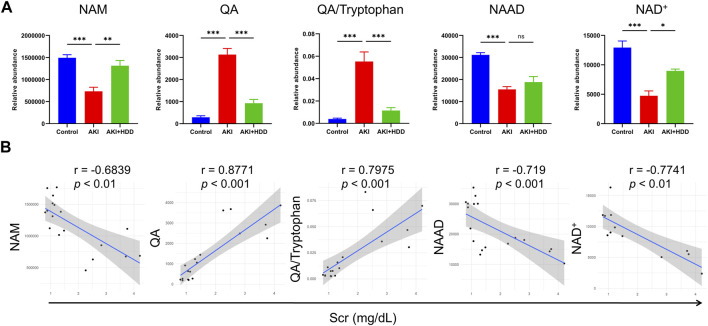
Levels and correlation analyses of metabolites related to NAD^+^ metabolism. **(A)** Relative abundance of NAM, QA, QA/Tryptophan, NAAD, and NAD^+^ in the control, AKI and AKI + HDD group. **(B)** Correlation analyses of NAM, QA, QA/Tryptophan, NAAD, and NAD^+^ with Scr. Data are expressed as mean ± SEM, *n* = 4-6 mice per group, one-way ANOVA followed by Tukey’s *post hoc* test was used for calculating statistical differences, ^*^ *p* < 0.05, ^**^ *p* < 0.01, ^***^ *p* < 0.001, ns, not significant.

We then focused on metabolites associated with nicotinate and nicotinamide metabolism because this pathway showed the highest enrichment and had been reported to participate in various kidney diseases (Ralto et al., 2020). Levels of nicotinamide (NAM), nicotinic acid adenine dinucleotide (NAAD), and NAD^+^ were lower in the kidney of AKI mice and could be significantly restored by HDD except for NAAD. Levers of quinolinic acid (QA) and QA/tryptophan in the *de novo* NAD^+^ synthesis pathway were markedly increased in AKI mouse kidneys and were normalized by HDD treatment (*p* < 0.001, Figure 7A). Correlation analysis showed that NAM, NAAD, and NAD^+^ were negatively correlated with Scr (*p* < 0.01), while QA and QA/tryptophan were positively correlated with Scr (*p* < 0.001, Figure 7B). These data suggested that renal NAD^+^ metabolism in AKI mice was disturbed and associated with renal function, which could be corrected by HDD treatment.

### HDD regulated the expression of enzymes involved in NAD^+^ synthesis in AKI mice

**FIGURE 8:**
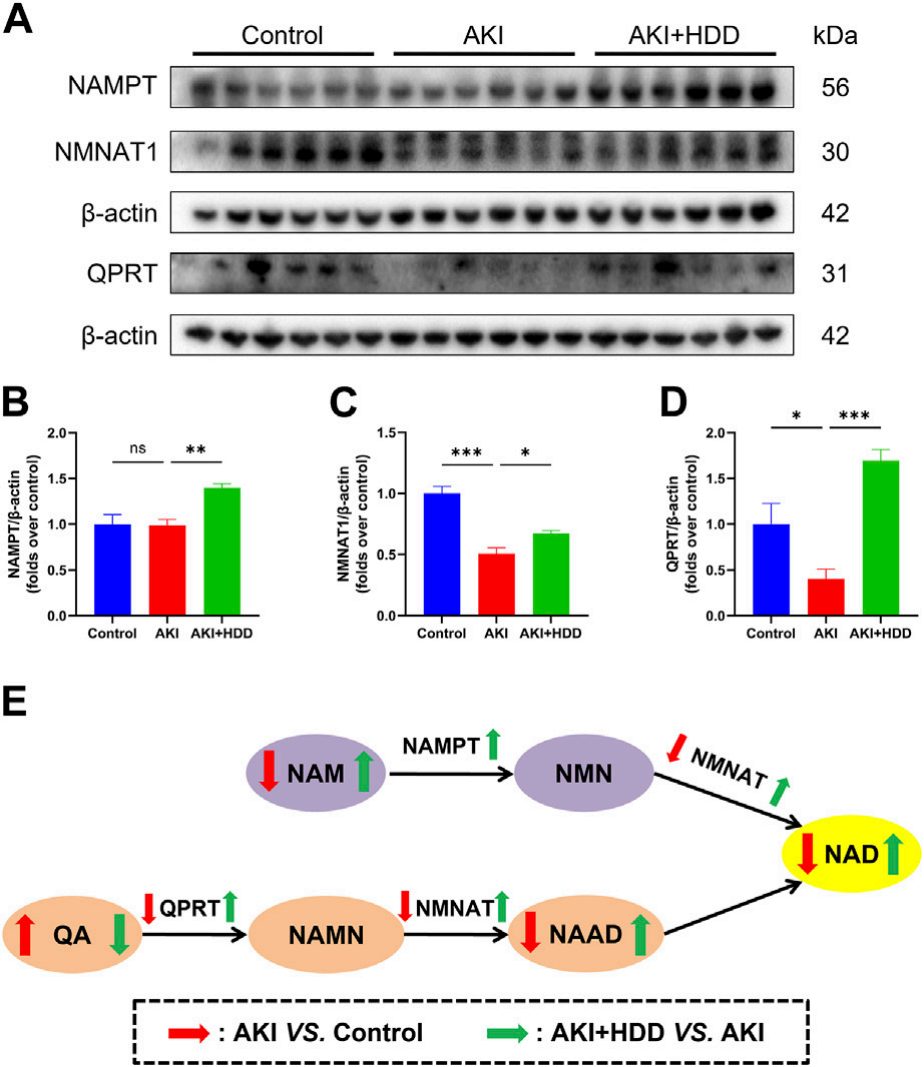
Effects of HDD on the expression of NAD^+^ biosynthesis-related enzymes in AKI mice. **(A)** Representative Western blot images of NAMPT, QPRT, and NMNAT1 expression in the kidneys of each group. **(B–D)** Densitometric analysis of NAMPT, QPRT, and NMNAT1 expression, normalized to β-actin. **(E)** Summary of NAD^+^ biosynthesis-related metabolites content and enzymes expression. Data are expressed as mean ± SEM, *n* = 6 mice per group, one-way ANOVA followed by Tukey’s *post hoc* test was used for calculating statistical differences, ^*^ *p* < 0.05, ^**^ *p* < 0.01, ^***^ *p* < 0.001, ns, not significant.

NAMPT, QPRT, and NMNAT are key enzymes for NAD^+^ synthesis (Zapata-Pérez et al., 2021). Western blot found that the expression of QPRT and NMNAT1 were all downregulated in the kidney of AKI mice (*p* < 0.05). Administration of HDD partially restored the expression of these two enzymes. Although no significant difference was observed in NAMPT expression between the AKI kidney and the control, HDD treatment significantly upregulated NAMPT expression in AKI mice (*p* < 0.01, Figures 8A–D). Summary of NAD^+^ biosynthesis-related metabolites content and enzymes expression was showed in Figure 8E.

## Discussion

In the present study, we explored whether HDD had a reno-protective effect on cisplatin-induced AKI, as well as its underlying mechanism. The results showed that HDD protected against cisplatin-induced renal dysfunction and tubular injury and suppressed apoptosis, inflammation, and oxidative stress in the kidney of AKI mice. Non-target metabolomics revealed that HDD regulated renal metabolite profiles in AKI mice, with a primary focus on nicotinate and nicotinamide metabolism. Furthermore, HDD administration was found to effectively restore the levels of NAD^+^ biosynthesis-related metabolites and enzymes expression in the kidney of AKI mice.

NAD^+^ is referred to as an “electron carrier” due to its capacity for accepting and donating electrons in cellular oxidation-reduction reactions (White and Schenk, 2012). This process is necessary for adenosine triphosphate (ATP) synthesis involved in cellular energy metabolism (Morevati et al., 2022). The kidney necessitates a substantial amount of energy to execute reabsorption function, and it is inherently one of the organs with the highest demand for NAD^+^ content (Zapata-Pérez et al., 2021). Apart from its function as an essential cofactor in energy metabolism, NAD^+^ also acts as a co-substrate for NAD^+^-consuming enzymes (Ralto et al., 2020; Zapata-Pérez et al., 2021; Morevati et al., 2022). The sirtuins (SIRTs), a family of NAD^+^-dependent histone deacetylases, are implicated in various physiological and pathological processes, including energy metabolism, aging, mitochondrial biogenesis, inflammation, DNA repairment, and stress resistance, etc .,(Vachharajani et al., 2016; Majeed et al., 2021; Rasti et al., 2023; Xu et al., 2023). The protective effects of SIRT1, SIRT3, and SIRT5 against cisplatin-induced kidney injury have been reported (Hasegawa et al., 2010; Kim et al., 2011; Morigi et al., 2015; Li W. et al., 2019). SIRT1 indirectly modulates peroxisome proliferator-activated receptor-γ coactivator-1-α (PGC-1α), whose diminished activity in kidney increases susceptibility to AKI (Fontecha-Barriuso et al., 2020). Pathologically activated NAD^+^-consuming poly (ADP-ribose) polymerases (PARPs) are implicated in renal ischaemia-reperfusion injury (IRI) (Martin et al., 2000), as evidenced by the increased resistance to renal IRI observed in *Parp1*-knockout mice (Zheng et al., 2005) and the protective effect against IRI provided by a PARP1 inhibitor (Ralto et al., 2020). NAD^+^ depletion has been observed in both cisplatin-induced and IRI models (Guan et al., 2017; Katsyuba et al., 2018). The reduction of renal NAD^+^ appears to inhibit certain reno-protective enzymes and trigger a series of reactions in the initiation and progression of AKI. Evidence to date suggests that AKI prognosis may be related to the capacity of NAD^+^ replenishment to buffer against pathological stress induced by reduced NAD^+^ levels (Ralto et al., 2020). In mammals, NAD^+^ is synthesized through three pathways: the *de novo* pathway from tryptophan, the salvage pathway from nicotinamide (NAM) and nicotinamide riboside (NR), and the Preiss-Handler pathway from nicotinic acid (NA) (Zapata-Pérez et al., 2021). Of note, impaired *de novo* NAD^+^ biosynthesis pathway was found in AKI (Poyan Mehr et al., 2018). The research also proposed that elevated urinary quinolinate/tryptophan could serve as an indicator for predicting AKI and other adverse outcomes. Our findings were in line with these results, as we observed an increase in both QA and QA/tryptophan levels in the kidneys induced by cisplatin. Since QA only participates in NAD^+^ *de novo* synthesis after being catalyzed by QPRT and not in other metabolic reactions (Poyan Mehr et al., 2018), QA accumulation indicates a reduction in QPRT activity. Further results from Western blot confirmed that the expression of QPRT was reduced in kidney induced by cisplatin and HDD significantly restored QPRT expression, reversing the elevated QA and QA/tryptophan levels. Besides, reduced intracellular NAD^+^ contents and a decreased ratio of NAD^+^: NADH were observed in cisplatin-treated renal tissue, resulting in reactive oxygen species (ROS) accumulation, inflammation, and kidney injury (Oh et al., 2014). NAM, a precursor of NAD^+^, exhibits potent antioxidant properties and can significantly mitigate the damage caused by ROS production under oxidative stress (Mejía et al., 2018). In our study, we observed a significant reversal of reduced levels of NAM and NAD^+^ in the kidneys of AKI mice with HDD treatment. NMNAT1 and NAMPT, two important enzymes in NAD^+^ synthesis pathways, were also upregulated upon HDD treatment.

Due to the potent nephrotoxicity of cisplatin, its clinical application as an anticancer agent for solid tumors is considerably restricted (Tang et al., 2023). Given that cisplatin is excreted through the kidneys, high doses or prolonged exposure to cisplatin leads to its accumulation in renal tubular cells (Zhang et al., 2021). This accumulation triggers diverse cellular stress responses, including apoptosis, inflammation, oxidative stress, DNA damage, and mitochondrial pathology (Wei et al., 2007; Basu and Krishnamurthy, 2010; Tang et al., 2021; Tang et al., 2023). Macrophages have critical roles in renal inflammation and repair. Our results found obvious macrophage infiltration in the kidneys of AKI mice and could be alleviated by HDD treatment (Figure 3). Macrophages are highly heterogeneous and can differentiate into two phenotypes, pro-inflammatory M1 macrophages and anti-inflammatory M2 macrophages. Previous studies have indicated that macrophage polarization played essential roles in renal fibrosis and folic acid nephropathy (Jiao et al., 2021a; Jiao et al., 2021b; An et al., 2023). However, the phenotype of macrophages and their response to HDD treatment in the present study need further investigation. NAD^+^ plays a crucial role in anti-aging, anti-inflammation, anti-oxidation, and DNA repair processes (Hershberger et al., 2017; Zapata-Pérez et al., 2021). The NAD^+^ precursor, NAM, has been reported to enhance mitochondrial metabolism and reduce ROS, implying that NAM supplementation may be a potential reno-protective approach against cisplatin nephrotoxicity (Tran et al., 2016). Combined with the results of our experiment, we have discovered that HDD could significantly restore NAM levels in cisplatin-induced kidney. Taken together, HDD may exert reno-protective effects against cisplatin-induced AKI through restoring NAD^+^ biosynthesis and thus replenish NAD^+^ content, thereby promoting a cascade of responses.

Although targeting NAD^+^ biosynthesis has been found to confer reno-protection (Allison, 2019) and improve health (Katsyuba et al., 2018), its effectiveness is limited due to the lack of renal targeting ability (Duan et al., 2023). It has been reported that natural products can modulate NAD^+^ homeostasis and exhibit various biological activities, including anti-apoptosis, anti-inflammation, anti-oxidation, improving energy metabolism, and neuroprotection. And most of them increase the content of NAD^+^, which is for preventing and treating diseases (Guo et al., 2023). Huangqi-Danshen, a classic combination of CHM, exerts invigorating effects on qi, promotes blood circulation, and facilitates detoxification. A plethora of studies have revealed that both Huangqi and Danshen possess beneficial improvements on diverse diseases by inhibiting inflammatory response, reducing cell apoptosis, alleviating oxidative stress, and activating immunomodulation (Ko et al., 2005; Qin et al., 2012; Shahrajabian et al., 2019; Li R. et al., 2023; Luo et al., 2023; Yang et al., 2023). Several components of Danshen have been reported to target NAD^+^ metabolism for therapeutic effects. Salvianolic acid B reversed the depletion of cellular NAD^+^ induced by angiotensin II to protect cardiomyocyte (Liu et al., 2014). Apigenin could increase NAD^+^ level and suppress neurodegeneration (Roboon et al., 2021) and protect against obesity and metabolic syndrome (Escande et al., 2013). The regulation of NAD^+^ metabolism by these components in models of kidney disease has not been reported. TCM adheres to the concept of disease prevention should take priority over disease onset. The results of our present study implied that HDD pretreatment could effectively counteract the nephrotoxic side effects induced by cisplatin to a certain extent, which was in line with the concept of disease prevention. However, the downstream effects of enhancing NAD^+^ availability and precise mechanisms by which HDD modulates NAD^+^ metabolism remain unclear and necessitate further investigation.

## Conclusion

In summary, HDD exerted a protective effect against cisplatin-induced AKI and suppressed apoptosis, inflammation, and oxidative stress in the kidney of AKI mice, which may be attributed to the modulation of NAD^+^ biosynthesis.

## Data Availability

The datasets presented in this study can be found in online repositories. The link to the repository and accession number can be found below: https://www.ebi.ac.uk/metabolights - MTBLS8876.

## Abbreviations

AKI, Acute kidney injury; ATP, Adenosine triphosphate; BUN, Blood urea nitrogen; CHM, Chinese herbal medicine; CKD, Chronic kidney disease; NA, Nicotinic acid; NAD+, Nicotinamide adenine dinucleotide; NAM, Nicotinamide; NAMPT, Nicotinamide phosphoribosyltransferase; NGAL, Neutrophil gelatinase-associated lipocalin; NMN, Nicotinamide mononucleotide; NMNAT1, Nicotinamide mononucleotide adenylyltransferase 1; NR, Nicotinamide riboside; QPRT, Quinolinate phosphoribosyltransferase; ROS, Reactive oxygen species; Scr, Serum creatinine; TCM, Traditional Chinese medicine.
