# Supplementary material for: Huangqi-Danshen decoction protects against cisplatin-induced acute kidney injury in mice
Source: Front Pharmacol. 2023 Nov 16;14:1236820. doi: 10.3389/fphar.2023.1236820 (PMC10687478; doi:10.3389/fphar.2023.1236820)
Supplement: Supplementary file 1 [file DataSheet1.PDF]

Table 1. The 165 altered metabolites in AKI mice significantly restored by HDD

No.	Name	m/z	Rt (s)	Mean Value			*P* Value		Fold Change	
				Control	AKI	AKI+HDD	AKI *VS.* Control	AKI+HDD *VS.* AKI	AKI *VS.* Control	AKI+HDD *VS.* AKI
1	(-)-Riboflavin	341.12	46.77	5462.98	2410.46	3562.29	3.08E-03	2.84E-02	0.44	1.48
2	(R)-(+)-Citronellic acid	188.16	183.40	1695.24	621.61	1537.94	6.68E-04	1.57E-02	0.37	2.47
3	.beta.-Cyano-L-alanine	175.07	203.26	10310.14	65995.56	25075.07	6.97E-05	7.81E-04	6.40	0.38
4	1,2-Benzenedicarboxylic acid	165.02	123.49	8981.46	75861.39	23898.10	8.77E-07	1.68E-05	8.45	0.32
5	12-Oxo-2,3-dinor-10,15-phytodienoic acid	323.18	269.45	3216.82	1546.70	2865.60	1.89E-03	3.19E-02	0.48	1.85
6	1-Methylnicotinamide	137.07	313.75	120567.05	312558.28	203531.50	1.26E-03	1.77E-02	2.59	0.65
7	1-O-(cis-9-Octadecenyl)-2-O-acetyl-sn-glycero-3-phosphocholine	1118.79	45.61	3940.23	2431.38	3701.40	3.97E-03	6.22E-03	0.62	1.52
8	1-Oleoyl-sn-glycero-3-phosphocholine	522.35	175.09	1377684.69	1141947.00	1267870.58	1.13E-02	3.28E-02	0.83	1.11
9	1-Phenylethylamine	144.08	182.74	7593.16	4770.08	6711.86	1.22E-02	3.71E-02	0.63	1.41
10	2(1H)-Pyridinone	96.04	61.96	123709.09	84549.04	113496.43	7.85E-05	4.05E-03	0.68	1.34
11	25-hydroxyvitamin D3	383.33	32.93	39803.17	21192.79	28521.26	5.22E-04	3.74E-02	0.53	1.35
12	2'-Deoxyadenosine 5'-monophosphate (dAMP)	376.03	83.18	265.97	3230.10	1134.56	3.02E-04	3.71E-03	12.14	0.35

13	2-Hydroxypyridine	78.03	61.96	288557.16	191544.54	265079.60	3.38E-04	6.06E-03	0.66	1.38
14	2-keto-D-Gluconic acid	210.06	68.92	1918.33	13705.13	7108.55	4.72E-05	4.24E-03	7.14	0.52
15	2-Methylguanosine	339.14	168.64	473.76	2459.19	1202.12	6.27E-06	9.39E-04	5.19	0.49
16	2'-O-methylcytidine	324.06	24.71	8343.50	4447.72	6987.14	4.01E-06	1.20E-03	0.53	1.57
17	2'-O-methylinosine	324.14	231.32	36270.38	24272.59	39808.94	2.59E-02	9.20E-03	0.67	1.64
18	2-Phenylacetamide	288.18	171.97	9260.58	1644.80	3187.41	5.38E-05	3.58E-03	0.18	1.94
19	3-Butynoic acid	148.04	182.07	22403.44	8220.78	17623.24	7.52E-03	1.71E-03	0.37	2.14
20	3-Hexanone	137.03	158.98	16503.48	13561.65	17803.01	3.16E-02	9.00E-03	0.82	1.31
21	3-Hydroxyanthranilic acid	154.05	91.74	1943.29	36804.17	11520.75	4.42E-04	4.02E-03	18.94	0.31
22	3-Hydroxydodecanoic acid	197.15	46.47	139592.07	291542.21	219836.71	6.31E-04	4.48E-02	2.09	0.75
23	3-Methoxy-4-Hydroxyphenylglycol Sulfate	263.02	39.16	160694.92	1905037.66	963736.44	2.75E-05	3.69E-03	11.85	0.51
24	3'-O-Methylinosine	281.09	141.42	2917.78	5130.52	4254.09	5.33E-05	2.88E-02	1.76	0.83
25	4-acetamidobutanoate	206.10	308.10	141665.34	48191.31	132828.51	1.11E-03	8.43E-05	0.34	2.76
26	4-Pyridoxic acid	184.06	39.28	2559.79	78313.15	23964.52	4.43E-06	1.74E-04	30.59	0.31
27	5(S),14(R)-Lipoxin B4	389.17	29.64	24682.40	8798.33	16079.99	4.82E-02	9.34E-03	0.36	1.83
28	5,6,7,8-tetrahydro-2-Naphthoic Acid	157.07	181.98	844.60	4004.72	2202.46	1.71E-04	1.00E-02	4.74	0.55
29	5-methoxyuridine	274.08	158.56	12956.53	6362.70	12197.68	8.69E-04	8.02E-04	0.49	1.92
30	5-Methyl-5,6-Dihydrouracil	146.09	304.31	753.11	5137.80	1093.86	2.40E-03	4.31E-03	6.82	0.21
31	Acetoacetic acid	120.06	68.28	6506.77	3284.08	4898.87	8.60E-04	3.85E-02	0.50	1.49
32	Acetohydroxamic acid	74.02	164.75	13620.15	101998.70	63112.57	2.20E-04	3.28E-02	7.49	0.62

33	Acetylcholine	146.12	187.63	7716.77	2357.73	4212.08	6.86E-06	8.77E-04	0.31	1.79
34	Acetyl-DL-Valine	319.19	347.07	793.11	3785.62	2419.19	1.46E-05	1.98E-02	4.77	0.64
35	Acetylglycine	233.08	299.32	2155.10	1008.99	1918.05	1.18E-05	1.05E-04	0.47	1.90
36	Adenine	136.06	149.80	1495076.14	490357.82	851024.43	6.71E-07	2.26E-02	0.33	1.74
37	Ala-Gly	207.09	308.10	67259.64	22961.43	63533.05	1.23E-03	1.25E-04	0.34	2.77
38	all cis-(6,9,12)-Linolenic acid	277.21	43.02	1712879.43	2831622.17	2017210.16	8.08E-04	1.92E-02	1.65	0.71
39	Allantoin	157.04	172.68	40502.34	350865.40	113678.30	1.08E-05	1.08E-04	8.66	0.32
40	Anthranilic acid (Vitamin L1)	137.04	323.45	23707.51	18227.19	31560.53	1.97E-02	1.56E-03	0.77	1.73
41	Arachidonoyl ethanolamide	348.29	35.38	19699.73	9119.96	18199.59	3.99E-07	6.46E-05	0.46	2.00
42	Arbutin	293.07	204.69	7902.35	5280.75	7561.42	6.66E-06	4.10E-03	0.67	1.43
43	Arg-Ala	245.15	233.96	4870.75	2455.24	3938.56	9.51E-04	4.49E-04	0.50	1.60
44	Arg-Glu	304.16	425.48	58291.42	49354.30	60461.61	2.51E-02	5.28E-03	0.85	1.23
45	Arg-Val	350.10	396.39	4908.11	3131.58	5733.76	2.12E-02	1.50E-02	0.64	1.83
46	Asn-Pro	252.10	98.57	24284.60	8840.91	17865.08	9.54E-06	1.71E-03	0.36	2.02
47	Azacitidine	267.07	148.80	71046.89	45339.40	74317.68	8.73E-03	1.92E-02	0.64	1.64
48	Baclofen	255.09	289.05	4303.06	17875.96	7149.78	2.79E-03	1.65E-02	4.15	0.40
49	Betaine	118.09	256.16	9411197.23	4895434.86	8408450.55	3.43E-06	6.14E-04	0.52	1.72
50	Codeine	317.19	419.38	16202.73	11740.16	17304.14	1.43E-02	4.38E-03	0.72	1.47
51	Coumarin	147.04	281.29	11217.37	7427.67	10152.78	4.47E-03	2.44E-03	0.66	1.37
52	Creatinine	114.06	159.39	821330.43	3490992.75	2571861.25	1.15E-08	1.12E-05	4.25	0.74
53	Crotonic acid	190.10	204.71	993.81	11977.52	6027.37	4.97E-04	2.58E-02	12.05	0.50

54	Cytidine 2',3'-cyclic phosphate	306.05	296.65	726.22	2484.98	1272.58	1.65E-04	5.59E-03	3.42	0.51
55	Cytidine 5'-diphosphocholine (CDP-choline)	489.11	415.84	53889.45	70965.42	45399.93	2.76E-02	8.68E-04	1.32	0.64
56	Cytidine 5'-monophosphate	324.06	419.63	28035.96	48622.25	38314.36	7.44E-06	3.33E-02	1.73	0.79
57	Cytidine 5'-monophosphate (CMP)	346.04	419.77	6930.17	11474.81	8332.91	2.84E-05	3.51E-03	1.66	0.73
58	D-Aspartic acid	134.04	375.63	36844.35	21820.00	26811.68	2.19E-04	1.63E-02	0.59	1.23
59	Deoxyinosine	294.12	83.96	17512.78	7743.32	14609.79	7.60E-08	8.10E-03	0.44	1.89
60	Dexpanthenol	221.15	46.19	55632.79	113714.68	82003.61	6.22E-05	6.97E-03	2.04	0.72
61	D-Glucuronate	159.03	204.51	160742.40	139443.25	166652.47	4.46E-02	2.83E-02	0.87	1.20
62	Dihydro-4,4-dimethyl-2,3-furandione	189.07	151.90	4652.87	1906.63	3835.11	1.80E-04	9.21E-03	0.41	2.01
63	Dihydroxyacetone	71.01	373.90	5826.66	3896.20	5553.01	1.32E-03	2.14E-02	0.67	1.43
64	DL-2-Aminoadipic acid	160.06	379.80	38649.16	106586.03	59936.56	2.03E-04	1.12E-02	2.76	0.56
65	DL-Homocysteine	118.03	289.95	4067.64	1675.11	4471.23	3.53E-03	2.52E-04	0.41	2.67
66	DL-Homoserine	161.09	98.88	7832.49	3225.60	5539.21	3.17E-04	7.52E-03	0.41	1.72
67	DL-lactate	89.02	216.60	2119124.67	1198235.55	1701317.64	2.27E-03	7.13E-03	0.57	1.42
68	D-Ribose	149.05	102.75	24338.69	161870.91	38746.06	1.30E-02	2.23E-02	6.65	0.24
69	D-Ribulose 5-phosphate	211.00	269.40	3352.49	5948.98	4449.40	8.21E-04	3.38E-02	1.77	0.75
70	Duloxetine	262.11	87.76	2427.55	20827.24	11129.19	5.82E-05	1.20E-02	8.58	0.53
71	Ethyl 3-hydroxybutyrate	196.09	91.47	4975.76	10883.31	3824.67	8.86E-03	3.36E-03	2.19	0.35

72	Fexofenadine	1003.56	180.12	2023.02	1132.92	1790.50	1.36E-03	7.44E-03	0.56	1.58
73	Flavin adenine dinucleotide (FAD)	786.15	367.15	26582.70	15901.96	22105.16	8.74E-07	2.34E-02	0.60	1.39
74	Flavone	223.08	61.96	17500.47	8765.48	14682.86	2.47E-04	1.85E-03	0.50	1.68
75	Floxuridine	246.06	23.44	1135.42	2709.65	1298.52	2.26E-02	4.74E-02	2.39	0.48
76	Glucosaminic acid	254.09	373.25	2372.66	1510.19	2388.43	2.30E-03	3.22E-03	0.64	1.58
77	Glycerol	151.06	107.84	2413.31	1737.43	2241.48	8.71E-04	2.12E-02	0.72	1.29
78	Glycylproline	155.08	324.46	2987.24	12648.76	3202.01	5.96E-03	6.86E-03	4.23	0.25
79	Guanosine	284.10	246.58	319481.59	157022.09	228438.95	1.33E-08	2.96E-03	0.49	1.45
80	His-Pro	235.12	134.56	1942.93	7307.33	3834.93	6.70E-06	7.14E-04	3.76	0.52
81	His-Thr	257.12	320.32	9696.54	6248.24	7706.87	3.04E-04	2.13E-02	0.64	1.23
82	Homovanillic acid	382.14	204.84	3134.84	6399.04	5403.20	3.92E-06	4.95E-02	2.04	0.84
83	Hydroxyhydroquinone	125.02	373.25	14454.08	10994.88	15348.51	1.03E-02	1.25E-02	0.76	1.40
84	Hypoxanthine	137.04	158.75	11115368.64	7400200.38	11557547.92	2.94E-03	7.97E-04	0.67	1.56
85	Ile-Thr	274.17	462.48	15823.64	8881.71	11252.40	1.67E-04	3.55E-02	0.56	1.27
86	Imidazole	110.07	291.19	2899.81	7548.02	3787.78	1.01E-03	7.86E-03	2.60	0.50
87	Imidazoleacetic acid	127.05	324.24	6891.41	9291.13	5472.49	3.72E-02	2.13E-03	1.35	0.59
88	Indole-2-carboxylic acid	144.05	43.81	3524.64	1471.74	3827.01	3.26E-02	3.91E-05	0.42	2.60
89	Indole-3-carboxylic acid	160.04	38.53	10590.57	35029.57	6339.98	2.09E-03	4.90E-04	3.31	0.18
90	Indolelactic acid	204.06	147.54	5681.99	48014.05	9776.41	2.17E-02	3.45E-02	8.45	0.20
91	Indoxyl sulfate	213.01	25.56	403238.59	3157762.06	1205712.54	1.41E-05	6.87E-04	7.83	0.38
92	Inosine	269.09	204.56	1517115.63	907264.40	1321632.10	1.12E-05	3.97E-04	0.60	1.46

93	Isovalerylglycine	158.08	177.14	73871.85	223226.84	138204.78	5.84E-05	3.54E-03	3.02	0.62
94	Isoxanthopterin	216.00	150.63	1476.39	4135.73	1062.78	2.87E-03	1.20E-03	2.80	0.26
95	Kynurenic acid	188.03	166.22	3700.52	53743.00	14353.83	5.19E-07	7.55E-06	14.52	0.27
96	L-Asparagine	133.06	251.16	1743.42	6857.69	1275.38	2.10E-02	1.35E-02	3.93	0.19
97	L-Carnitine	162.11	372.19	1404407.72	797054.08	1103376.61	1.62E-02	4.12E-02	0.57	1.38
98	Leu-Val	231.17	356.72	8893.22	18582.11	15269.35	6.34E-06	1.28E-02	2.09	0.82
99	Linalool oxide	231.16	282.10	759.68	3660.13	1717.32	7.51E-04	1.06E-02	4.82	0.47
100	Linoleoyl ethanolamide	324.29	35.75	31116.56	20580.26	34469.07	4.59E-04	3.88E-04	0.66	1.67
101	L-Isoleucine	130.09	268.89	138641.77	109026.42	162077.03	3.05E-02	1.24E-03	0.79	1.49
102	L-Methionine	148.04	179.57	9371.86	4396.86	7734.49	5.84E-04	1.51E-03	0.47	1.76
103	L-Saccharopine	277.14	420.36	51341.98	142720.96	67384.00	1.47E-04	1.49E-03	2.78	0.47
104	L-Serine	106.05	355.87	7623.76	5444.55	7638.48	5.62E-03	2.25E-03	0.71	1.40
105	Lumichrome	243.09	51.04	398127.85	187863.51	281177.82	3.59E-06	9.77E-03	0.47	1.50
106	Lys-Cys	214.10	421.02	4578.28	13918.70	6608.46	2.66E-04	3.02E-03	3.04	0.47
107	Lys-His	328.14	205.35	66099.25	51347.69	66612.51	1.77E-03	3.69E-03	0.78	1.30
108	Lys-Phe	316.16	470.70	11050.18	6237.65	8184.44	3.09E-05	4.44E-02	0.56	1.31
109	Malonic acid	103.00	375.64	13284.71	40777.71	23194.59	7.82E-04	1.81E-02	3.07	0.57
110	Met-Ala	203.08	322.33	43758.86	11591.29	40307.89	1.66E-03	8.94E-04	0.26	3.48
111	Methoprene (S)	311.25	39.57	9027.42	33757.94	20466.37	3.17E-05	8.13E-03	3.74	0.61
112	Methoxyacetic acid	151.06	143.70	9119.87	58611.94	42570.10	1.19E-07	2.21E-02	6.43	0.73
113	Methylthiouracil	158.04	172.67	2039.62	16816.00	5480.72	3.24E-05	2.83E-04	8.24	0.33
114	Muramic acid	288.05	31.30	734.35	8056.61	1767.98	1.05E-05	7.51E-05	10.97	0.22
115	N1-Methyl-2-pyridone-5-carboxamide	153.06	87.74	32440.42	1299677.58	351316.27	7.33E-04	6.34E-03	40.06	0.27
116	N4-Acetylcytidine	286.10	155.06	2116.82	6398.54	4907.47	2.79E-09	2.93E-03	3.02	0.77

117	N6-Methyladenine	337.10	107.27	23391.73	12106.42	16537.64	3.04E-03	4.73E-02	0.52	1.37
118	N-Acetyl-D-lactosamine	384.15	349.87	2512.44	5501.74	3781.85	1.06E-05	2.76E-03	2.19	0.69
119	N-Acetyl-DL-methionine	190.05	179.54	95635.17	36353.89	81322.35	2.71E-04	1.40E-04	0.38	2.24
120	N-Acetyl-L-alanine	130.05	233.65	21649.72	10980.89	20141.65	1.09E-02	7.80E-04	0.51	1.83
121	N-Acetyl-L-methionine	192.07	180.47	1963.27	1280.05	2096.98	6.25E-03	3.65E-03	0.65	1.64
122	N-Acetylmannosamine	202.07	143.55	4242.61	9099.18	6597.91	1.73E-06	4.16E-06	2.14	0.73
123	N-Carboxyethyl-.gamma.-aminobutyric acid	212.04	175.56	882.25	2498.81	1398.23	2.84E-05	1.40E-03	2.83	0.56
124	NG,NG-dimethyl-L-arginine(ADMA)	203.15	487.90	274139.40	214720.00	268486.57	4.18E-03	1.25E-02	0.78	1.25
125	N-Glycolylneuraminic acid	326.10	375.64	9367.45	22717.74	11954.04	2.06E-03	1.43E-02	2.43	0.53
126	Nicotinamide	123.05	95.66	1493970.54	734420.19	1313891.79	6.15E-05	3.25E-03	0.49	1.79
127	Nicotinuric acid	378.13	315.51	2068.16	3976.61	2649.78	2.28E-04	2.89E-03	1.92	0.67
128	Nitrobenzene	264.10	25.13	1328.43	37030.35	2503.81	8.78E-06	1.20E-05	27.88	0.07
129	Nname,Cinoxacin	263.07	25.28	1935.37	9431.22	2184.86	3.21E-05	1.71E-06	4.87	0.23
130	N-Tigloylglycine	158.08	182.09	876.52	2110.61	1478.33	1.25E-05	2.74E-03	2.41	0.70
131	O-Acetyl-L-serine	148.06	42.17	3569.13	10009.66	5399.89	2.97E-06	9.53E-04	2.80	0.54
132	O-Succinyl-L-homoserine	256.03	34.51	21238.32	96402.28	48264.69	4.09E-07	1.56E-05	4.54	0.50
133	Oxyquinoline	146.06	180.13	1431.46	706.60	1081.93	1.08E-02	1.06E-02	0.49	1.53
134	Pantothenate	220.12	251.38	107102.33	71350.42	131879.94	1.57E-03	1.11E-05	0.67	1.85
135	p-Cresol	107.05	22.81	11171.99	1559897.62	529569.89	8.78E-12	3.04E-06	139.63	0.34

136	Perseitol	193.07	165.86	11274.04	47259.93	27837.34	2.76E-04	1.62E-02	4.19	0.59
137	Phenylacetic acid	119.05	281.34	12029.29	8185.45	11407.19	6.39E-03	5.37E-04	0.68	1.39
138	Phenylacetylglycine	192.07	165.51	31001.08	461774.69	248222.65	9.93E-05	1.20E-02	14.90	0.54
139	Phosphoenolpyruvate	188.96	417.75	14740.80	6021.35	9523.81	2.26E-03	2.24E-02	0.41	1.58
140	Pimelic acid	202.11	251.11	8752.51	6902.64	10603.17	4.18E-04	1.98E-05	0.79	1.54
141	Primidone	218.11	306.53	1035.42	4369.47	1859.03	8.28E-04	6.49E-03	4.22	0.43
142	Pro-Ala	187.11	316.06	26245.37	14469.63	20167.71	8.06E-04	1.04E-03	0.55	1.39
143	Propoxur	227.14	273.75	1398.54	3298.72	1927.04	6.00E-04	1.79E-03	2.36	0.58
144	Pro-Ser	266.11	355.09	28515.32	17457.14	23654.42	7.65E-03	4.96E-02	0.61	1.36
145	Pro-Thr	258.15	256.10	109497.46	41202.92	94743.55	1.45E-07	1.67E-04	0.38	2.30
146	Pyridoxamine 5'-phosphate	248.06	25.79	980.31	12535.77	3456.02	4.00E-05	4.05E-04	12.79	0.28
147	Quinaldic acid	191.08	45.58	17908.64	10132.23	18980.52	4.96E-04	2.83E-03	0.57	1.87
148	Quinolinate	231.03	26.13	291.20	3136.14	928.40	1.62E-06	4.92E-05	10.77	0.30
149	Rutin	669.16	246.31	2558.77	831.99	1275.92	2.03E-06	3.38E-03	0.33	1.53
150	Saccharin	242.01	172.53	1422.72	3325.14	2214.89	1.52E-04	9.34E-03	2.34	0.67
151	S-Adenosyl-L-homocysteine	383.12	281.34	2431.76	1194.85	1967.49	1.25E-03	7.26E-03	0.49	1.65
152	Salicyluric acid	194.04	217.39	1590.48	7727.90	2706.79	3.42E-06	2.17E-05	4.86	0.35
153	Sebacic acid	201.11	296.46	9573.97	28099.06	15890.71	3.58E-03	3.00E-02	2.93	0.57
154	Sildenafil	473.19	316.93	2189.54	1180.66	2267.31	1.08E-02	2.51E-03	0.54	1.92
155	Taurine	124.01	276.46	3595272.83	2975457.16	3752610.96	1.87E-02	8.74E-03	0.83	1.26
156	Thiamine	265.11	355.10	207530.10	133746.19	173330.65	3.46E-03	4.59E-02	0.64	1.30
157	Thiamine monophosphate	365.05	205.21	7019.32	5833.91	7216.77	2.18E-03	8.72E-03	0.83	1.24

158	Thr-Thr	221.12	251.36	10304.64	7286.75	12058.18	6.95E-03	9.18E-05	0.71	1.65
159	Trigonelline	138.05	269.59	39409.09	8798.95	16033.51	1.53E-04	1.38E-02	0.22	1.82
160	Tyr-Asp	296.10	107.42	10492.36	4176.00	6104.81	1.55E-05	1.28E-02	0.40	1.46
161	Uracil mustard	251.02	52.36	4720.94	27718.69	9096.78	1.65E-05	1.73E-04	5.87	0.33
162	Urocanic acid	174.99	22.96	20554.79	544046.03	184129.47	8.91E-08	2.13E-05	26.47	0.34
163	Vanylglycol	165.06	39.39	8180.86	15442.28	10866.65	4.51E-03	1.59E-02	1.89	0.70
164	Vindoline	437.21	251.28	3885.55	1821.52	5182.65	4.94E-04	4.74E-04	0.47	2.85
165	Xanthurenic acid	204.03	165.42	10827.06	23765.18	14263.92	5.82E-08	7.24E-06	2.19	0.60
