# Supplementary material for: Huangqi-Danshen decoction protects against cisplatin-induced acute kidney injury in mice
Source: Front Pharmacol. 2023 Nov 16;14:1236820. doi: 10.3389/fphar.2023.1236820 (PMC10687478; doi:10.3389/fphar.2023.1236820)
Supplement: Supplementary file 2 [file Image1.pdf]

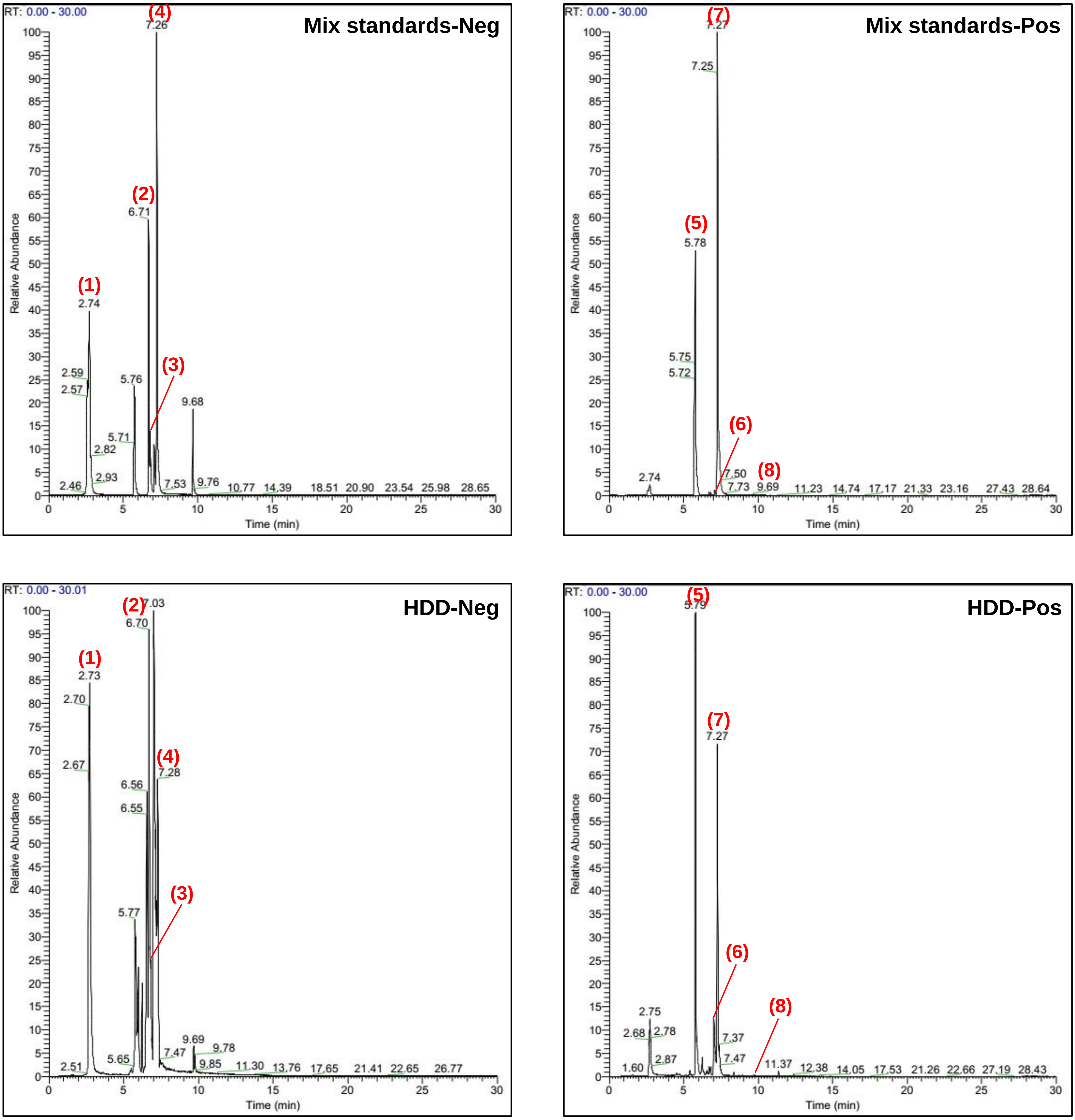

The peaks 1-8: (1) Protocatechualdehyde, (2) Rosmarinic Acid, (3) Lithospermic Acid, (4) Ononin, (5) Calycosin 7-O-beta-D-glucoside, (6) Salvianolic Acid B, (7) Daidzein, (8) Astragaloside IV.
